# Elevation of NO production increases Fe immobilization in the Fe-deficiency roots apoplast by decreasing pectin methylation of cell wall

**DOI:** 10.1038/srep10746

**Published:** 2015-06-15

**Authors:** Yi Quan Ye, Chong Wei Jin, Shi Kai Fan, Qian Qian Mao, Cheng Liang Sun, Yan Yu, Xian Yong Lin

**Affiliations:** 1 MOE Key Laboratory of Environment Remediation and Ecological Health, College of Natural Resource & Environmental Sciences, Zhejiang University, Hangzhou 310058, China; 2 Key Laboratory of Subtropical Soil Science and Plant Nutrition of Zhejiang Province, College of Environmental and Resource Sciences, Zhejiang University, Hangzhou 310058, PR China

## Abstract

Cell wall is the major component of root apoplast which is the main reservoir for iron in roots, while nitric oxide (NO) is involved in regulating the synthesis of cell wall. However, whether such regulation could influence the reutilization of iron stored in root apoplast remains unclear. In this study, we observed that iron deficiency elevated NO level in tomato (*Solanum lycopersicum*) roots. However, application of S-nitrosoglutathione, a NO donor, significantly enhanced iron retention in root apoplast of iron-deficient plants, accompanied with a decrease of iron level in xylem sap. Consequently, S-nitrosoglutathione treatment increased iron concentration in roots, but decreased it in shoots. The opposite was true for the NO scavenging treatment with 2-(4-carboxyphenyl)-4,4,5,5-tetramethylimidazoline-1-oxyl-3-oxide (cPTIO). Interestingly, S-nitrosoglutathione treatment increased pectin methylesterase activity and decreased degree of pectin methylation in root cell wall of both iron-deficient and iron-sufficient plants, which led to an increased iron retention in pectin fraction, thus increasing the binding capacity of iron to the extracted cell wall. Altogether, these results suggested that iron-deficiency-induced elevation of NO increases iron immobilization in root apoplast by decreasing pectin methylation in cell wall.

Iron (Fe) is an essential micronutrient for plant growth and acts as a cofactor of many enzymes required for essential physiological processes including photosynthesis and respiration. Although abundant in soil, its bioavailability is often limited for a variety of field crops around the world, due to its low solubility in soil, especially in well aerated calcareous soil^1^. Under Fe deficiency, plants have evolved several strategies to enhance Fe uptake from soil with limited Fe availability, including both morphological alterations like increased developments of sub-apical root hair and lateral roots, and physiological changes such as enhanced expression of Fe uptake components (i.e. Iron Regulated Ttransporter1 (IRT1) and Ferric Chelate Reductase (FCR) in nongraminaceous monocot and dicot plants, and Yellow Stripe Like (YSL) in graminaceous plants)^2^,^3^,^4^. In addition to the enhanced Fe uptake from rhizosphere by roots, our previous study identified that reutilization of Fe normally stored and unavailable in the root apoplast is also an important strategy for plant under Fe-limited condition^5^.

Cell wall is the major component of root apoplast, consisting of pectin, cellulose and hemicellulose, and containing highly negatively charged sites that may serve as a sink for most cationic nutrients^6^. It is estimated that at least 75% of the total Fe in roots is located in the root apoplast^7^. Therefore, the factors altering cell wall synthesis of roots should be expected to have an impact on Fe storage in apoplast as well as its subsequent reutilization by plants. Interestingly, several studies have shown that Fe deficiency would result in a quickly increase of NO production in roots^8^,^9^,^10^, while NO is a freely diffusible and reactive signal molecule, which has been identified to be involved in regulating synthesis of the cell wall. For example, exogenous application of NO affected cellulose content in roots of tomato plants in a dose dependent manner^11^. In addition, Xiong *et al.*^12^ found that exogenous supply of NO increased pectin and hemicelluloses content in rice roots. Since cell wall are full of negative charges, metal ions can be fixed into cell wall component, therefore the alteration of cell walls components could be expected to affect the binding capacity of metal ions with cell wall^6^. In this sense and taking account of the important role of root apolast in Fe homeostasis in plant, we assumed that elevation of NO production may affect the Fe retention in root apoplast and its subsequent reutilization by plant through a mechanism of modulating the composition of cell wall.

In this study, we used tomato plants and pharmacological treatments to test the above hypothesis. We found that Fe deficiency-induced NO production increased the Fe immobilization in the Fe-deficient root apoplastic space through lowering the degree of methyl esterification of cell wall, ultimately preventing Fe translocation from roots to shoots.

## Results

### Effect of Fe deficiency on the production of NO

**Figure 1:**
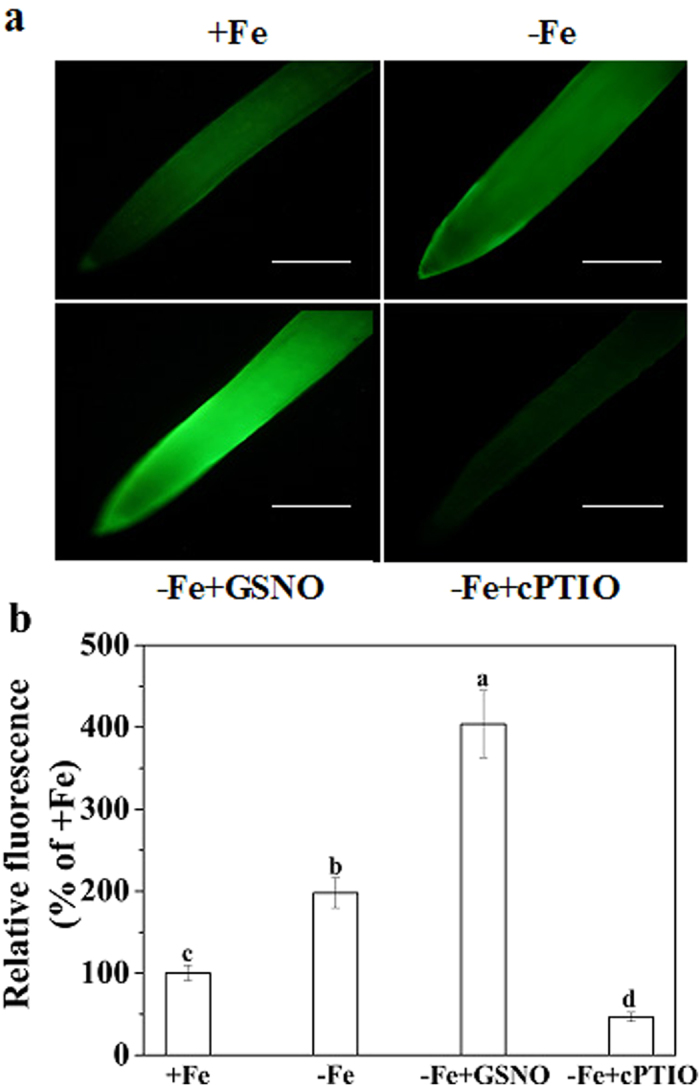
NO production in root of tomato plants treatment with NO donor or scavenger under Fe deficiency. The seedlings were grown under Fe sufficient (50 μM Fe) or Fe deficient (1 μM Fe) conditions treated with or without 100 μM GSNO or 100 μM cPTIO for 7 days. The roots of plants after 7 days of treatment were harvested for NO analysis. Photographs of NO production shown as green fluorescence in representative roots (**a**) (bar = 1 mm). NO production expressed as relative fluorescence (**b**) Data are means ± SD (n = 15). Bars with different letters are significantly different at *P* < 0.05.

The effect of Fe deficiency on NO production in roots was evaluated by using a NO fluorescence probe diaminofluorescein-FM diacetate (DAF-FM DA). As shown in Fig. 1a, Fe deficient treatment (1 μM Fe-EDTA) had a higher green fluorescence in roots than Fe sufficient treatment (50 μM Fe-EDTA). Based on the signal intensity of fluorescence, the NO content in roots of Fe deficient plants increased by about 98% as compared to the Fe sufficient plants (Fig. 1b). Application of the NO specific scavenger cPTIO substantially diminished the Fe-deficiency-induced NO-associated green fluorescence (Fig. 1a). These results suggested that Fe deficiency promoted NO production in roots of tomato plants.

### Effect of NO on Fe retention in root apoplastic space

**Figure 2:**
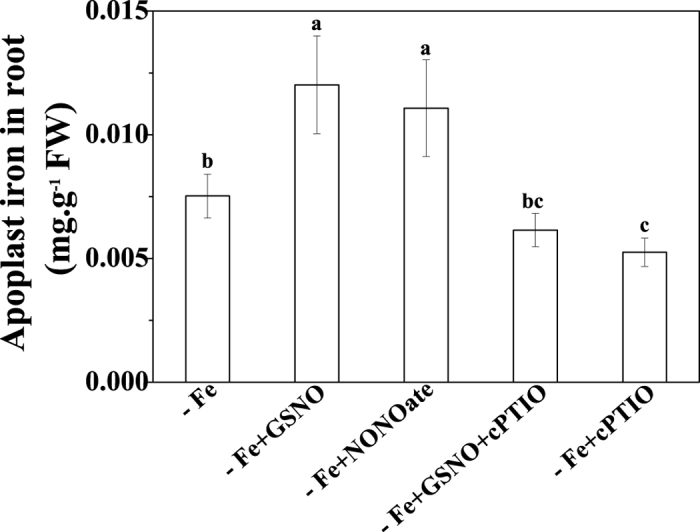
Effect of NO on Fe concentrations in root apoplast of tomato plant under Fe deficient conditions. The seedlings were grown under Fe deficient (1 μM Fe) conditions treated with either 100 μM GSNO, 100 μM NONOate, 50 μM cPTIO or 100 μM GSNO plus 50 μM cPTIO for 7 days and Fe concentrations in root apoplast were determined. Error bars represent ± SD (n = 4). Bars with different letters are significantly different at *P* < 0.05.

In view of the critical role of root apoplast in Fe homeostasis within plant, we tested whether the alteration of NO level had an impact on the Fe accumulation in root apoplast. Application of S-nitrosoglutathione (GSNO) in Fe-deficient growth medium significant increased Fe concentration in root apoplast (Fig. 2). Treatment with another NO donor, NONOate, showed a similar effect to the GSNO treatment. In contrast, application of NO scavenger cPTIO clearly decreased the Fe concentration in root apoplast (Fig. 2). We also used the Arabidopsis mutant *Atgsnor1-3*, which lost the function of GSNOR reductase and accumulated higher level of NO^13^, to clarify whether an alteration of endogenous NO levels in roots have an impact on Fe accumulation in root apoplast. As shown in Supplementary Fig. S1, under Fe-deficient conditions, the root apoplast of *Atgsnor1-3* contained more Fe than that of the wild type Arabidopsis *Col-0*. The above results indicated that Fe-deficency-induced NO production increased Fe immobilization in root apoplast. However, an opposite trend was observed under Fe-sufficient conditions, where the root apoplast Fe decreased significantly upon GSNO treatment as compared with controls (Supplementary Fig. S3).

### Effects of exogenous NO donor treatment on Fe homeostasis in plants

**Figure 3:**
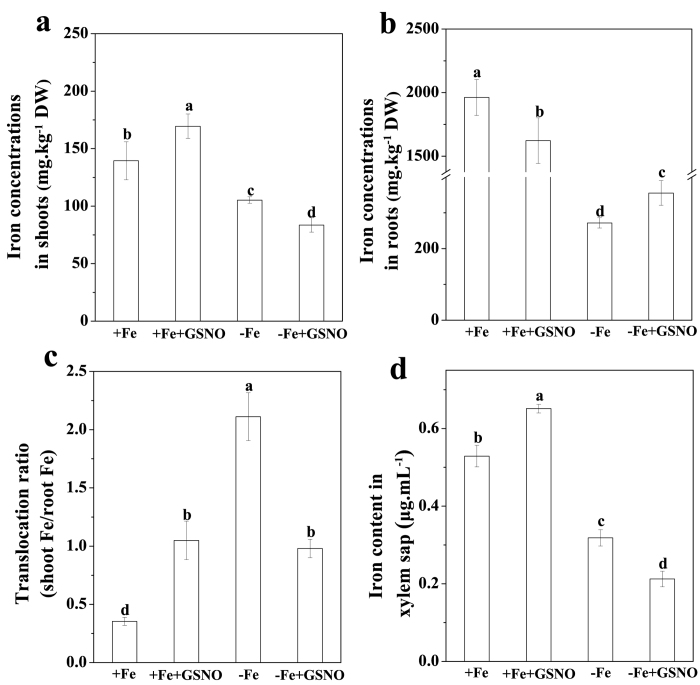
Effect of NO on the Fe concentrations, iron transport rate and xylem sap iron in tomato plant. The seedlings were treated with or without 100 μM GSNO for 7 days under Fe deficient conditions (1 μM Fe) or Fe sufficient condition (50 μM Fe), and then plants were harvested into root and shoot samples and analyzed for Fe concentrations. Shoot iron concentrations (**a**), root iron concentrations (**b**) root iron translocation ratio (**c**) and Fe in xylem sap (**d**) Error bars represent ± SD (n = 6). Bars with different letters are significantly different at *P* < 0.05.

To further investigate whether GSNO treatment altered Fe homeostasis within plant, we analyzed Fe concentrations in both shoots and roots of Fe-deficient plants. The GSNO treatment significantly decreased the Fe level in shoots, whereas the opposite was true in roots (Fig. 3a,b). The rate of total shoot Fe to total root Fe was calculated, and in the Fe-deficient plant, it was greatly suppressed by the GSNO treatment (Fig. 3c). In addition, the GSNO treatment also significantly decreased the Fe in xylem sap (Fig. 3d). However, different effects of NO on the Fe concentration in shoots, roots, xylem sap and the translocation rate of plants were observed under Fe-sufficient conditions (Fig. 3). These results suggested that GSNO affects Fe homeostasis in different manners under different Fe growth conditions.

### Effects of NO on the retention of Fe in root cell wall components

**Figure 4:**
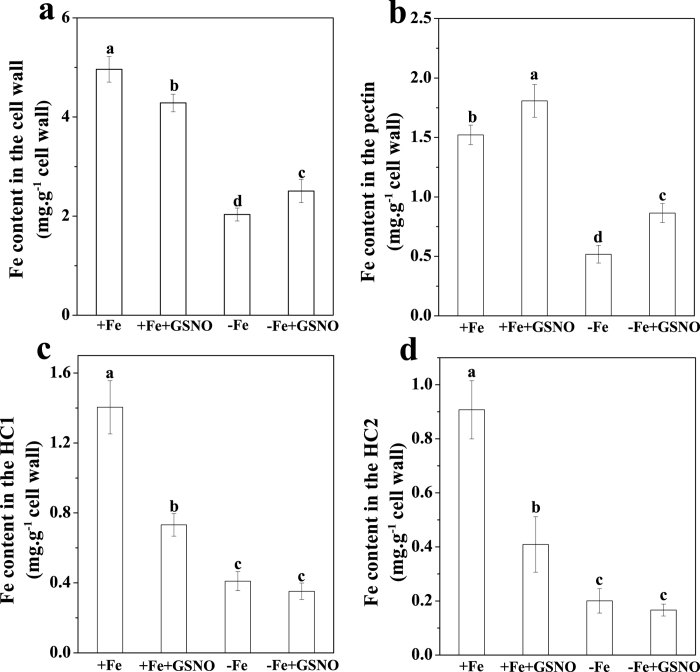
Effect of exogenous GSNO treatment on Fe content in root cell wall components. The seedlings were treated with or without 100 μM GSNO for 7 days under Fe deficient conditions (1 μM Fe) or Fe sufficient conditions (50 μM Fe), and then roots were harvested and cell wall extraction and fraction for Fe contents analyzed. Fe content in cell wall (**a**) Fe content in pectin (**b**) Fe content in HC1 (**c**) and Fe content in HC2 (**d**) Error bars represent ± SD (n = 4). Bars with different letters are significantly different at *P* < 0.05.

To further elucidate the contrasting effects of NO on Fe accumulation in roots, the Fe content in root cell wall was analyzed. We found that treatment with GSNO led to decreases in Fe content in cell wall under Fe-sufficient conditions, while the Fe content increased in cell wall under Fe-deficient conditions (Fig. 4a). Moreover, a significant increase in pectin Fe under both Fe-sufficient and Fe-deficient conditions was observed when treated with GSNO (Fig. 4b). For Fe contents in hemicellulose 1 (HC1) and hemicellulose 2 (HC2) (expressed as Fe-HC1 and Fe-HC2 respectively), an opposite trend was obtained when compared with the Fe content in pectin, i.e., the Fe contents in both HC1 and HC2 decreased by the GSNO treatment irrespective of Fe growth conditions (Fig. 4c,d). In addition, much greater decreases in Fe contents in HC1 and HC2 were found in Fe-sufficient plants than in Fe-deficient plants.

### Effects of NO on the synthesis of root cell wall and its Fe adsorption and desorption properties

**Figure 5:**
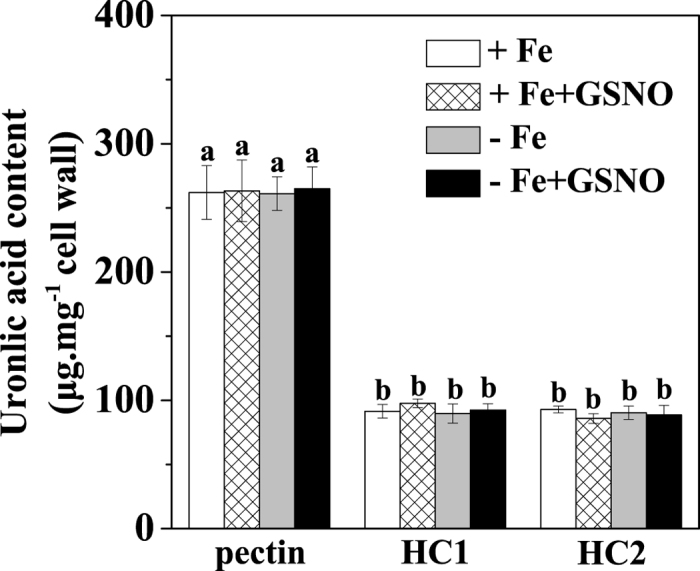
Effect of NO on the cell wall compositions in tomato roots. The seedlings were treated with or without 100 μM GSNO for 7 days under Fe deficient condition (1 μM Fe) or Fe sufficient condition (50 μM Fe), and roots were sampled and cell wall polysaccharides were fractionated into pectin, HC1, HC2 for uronic acid content measurement. Error bars represent ± SD (n = 4). Bars with different letters are significantly different at *P* < 0.05.

**Figure 6:**
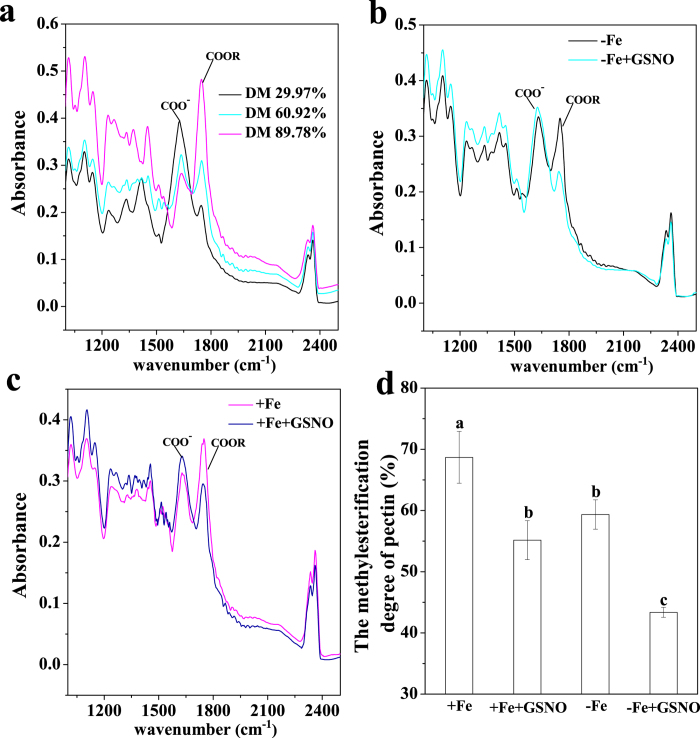
Effect of NO on the degree of methyl-esterification (DM) of the pectin. The seedlings were treated with or without 100 μM GSNO for 7 days under Fe deficient conditions (1 μM Fe) or Fe sufficient condition (50 μM Fe) and the DM of pectin were analyzed. FTIR spectra of standard pectins with different DM (**a**) the two peaks indicated conventionally used to determine the DM. FTIR spectrum of pectin from –Fe and –Fe+GSNO treatment (**b**) FTIR spectrum of pectin from +Fe and +Fe+GSNO treatment (**c**) The degree of methyl-esterification of the pectin from tomato root analyzed by FTIR (**d**) Error bars represent ± SD (n = 5). Bars with different letters are significantly different at P < 0.05.

**Figure 7:**
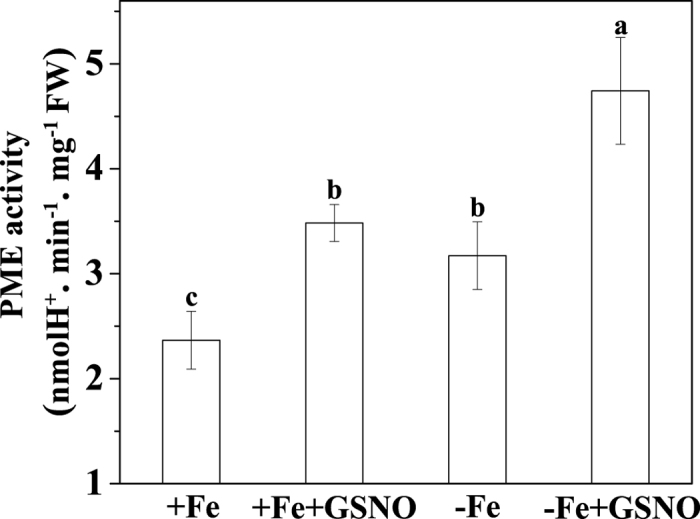
Effect of NO on the activity of pectin methylesterase (PME) in the root of tomato plant. The seedlings were treated with or without 100 μM GSNO for 7 days under Fe deficient conditions (1 μM Fe) or Fe sufficient conditions (50 μM Fe) and then the PME activity were analyzed. Error bars represent ± SD (n = 5). Bars with different letters are significantly different at *P* < 0.05.

**Figure 8:**
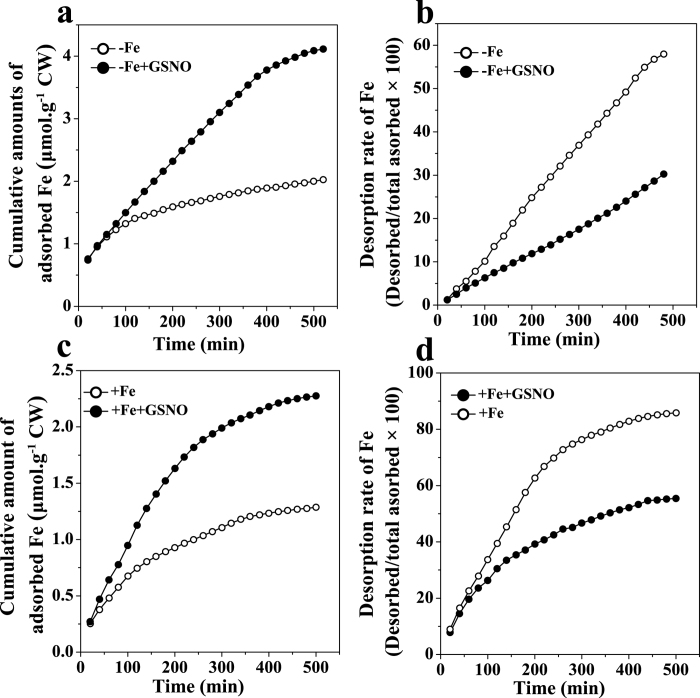
Adsorption (**a**,**c**) and desorption (**b**,**d**) kinetics of Fe in the root cell wall of tomato plant. The seedlings were exposed to Fe-deficient (1 μM Fe) solution (**a**,**b**) or Fe sufficient (50 μM Fe) conditions (**c**,**d**) treated with or without 100 μM GSNO for 7 days and then cell wall materials were extracted and analyzed.

Because NO has been implicated in the regulation of cell wall synthesis^11^, we further explored whether the exogenous GSNO treatment had an impact on the composition of root cell wall. As shown in Fig. 5, the content of pectin, HC1 and HC2 did not vary significantly after GSNO treatment under both Fe-deficient and Fe-sufficient conditions when compared to the control, suggesting that the NO-increased Fe accumulation in pectin should not be associated with an alteration of the three cell wall components. On the other hand, the degree of pectin methylation has been shown as another factor determining the binding capacity of metal ions with cell walls^14^. We first measured the degree of pectin methylation by FTIR spectroscopy, and found that GSNO significantly decreased the degree of pectin methylation, under both Fe-deficient and Fe-sufficient conditions (Fig. 6d). A similar result was also observed by using spectrophotometric measurement (Supplementary Fig. S4). In agreement with the changes in the degree of pectin methylation, GSNO treatment significantly increased the PME activity under both Fe-deficient and Fe-sufficient conditions (Fig. 7). Alteration of pectin methylation in cell wall may affect its Fe retention capacity. The adsorption kinetics analysis shown that cell wall from GSNO treatment had higher adsorption ability to Fe than the control (Fig. 8a). In contrast, the desorption ability was higher in the cell wall from the control treatment (Fig. 8b). A similar pattern of adsorption and desorption kinetics was also observed in the cell wall from Fe-sufficient conditions (Fig. 8c,d). This finding suggested that the lower degree of methyl esterification of pectin upon GSNO treatment led to an increased binding ability of cell wall with Fe.

## Discussion

As a crucial signal molecule in plants, NO has been shown to play a vital role in diverse physiological responses^15^,^16^,^17^,^18^. Increased accumulation of NO under Fe deficiency had been observed in various plants^8^,^9^,^19^. This was also true for tomato plants in our present study (Fig. 1). Interestingly, when the plant was exogenously treated with NO donor, GSNO or NONOate, which resulted in a further increase in the NO level in roots (Fig. 1; Supplementary Fig. S2), the level of Fe stored in the root apoplast was significantly enhanced under Fe deficiency. The opposite is true when the Fe-deficient plant treated with NO scavenger cPTIO (Fig. 2). The results suggested that Fe-deficiency-induced NO production increases Fe immobilization in the root apoplast under Fe deficiency. The above process should be expected to decrease Fe translocation from roots to shoots. This assumption was confirmed by calculating the Fe translocation rate, as the GSNO treatment clearly decreased the ratio of shoot Fe to root Fe (Fig. 3c). In addition, the Fe concentration in xylem sap of GSNO-treated plants was lower than that of control plants (Fig. 3d), providing further evidence for the above conclusion. Unexpectedly, here we observed an opposite effect of NO on Fe accumulation in root apoplast and Fe translocation from roots to shoots in Fe-sufficient plants (Supplementary Fig. S3; Fig. 3c). The possible mechanisms underlying the variance in the effect of NO on root apoplastic Fe accumulation under different Fe supplies will be discussed latter.

Reutilization of Fe in root is a strategy employed by plant under Fe deficiency. For instance, Jin *et al.*^5^ observed that restraint of Fe reutilization in root apoplast by removing the secreted phenolic compounds aggravated Fe-deficiency when the plants were grown under Fe-limited conditions. Recently, Pavlovic *et al.*^20^ found that promotion of Fe mobilization in root apoplast by silicon application could alleviate Fe deficiency in cucumber plants. Therefore, it seems that hampered the reutilization of apoplastic Fe by NO may be unfavorable for plant Fe nutrient. However, previous reports suggested that NO is involved in regulating Fe-deficient induced responses, including induction of the genes expression related to Fe acquisition and lateral root development^8^,^10^,^21^. These Fe-deficient induced responses have been proved to favor the plant Fe uptake. The reason of above paradoxical functions of NO in Fe nutrition is unclear. A possible explanation for this observation may be as bellows: the primary function of NO under Fe deficiency is to enhance the Fe-deficiency-induced responses and increase the uptake of Fe. Meanwhile, because of the signaling role of NO in regulating cell wall synthesis, the increased NO by Fe deficiency may unavoidably alter the composition of cell wall in roots, thus altering the capacity of Fe binding in the apoplast. In this sense, therefore, under Fe-deficient condition, the enhancement of Fe retention in root apoplast by elevated NO probably is a side-effect of NO which is originally used for regulating Fe-deficient responses. From this view of point, the function of NO seems to be similar as reactive oxygen species (ROS) which functions inducing defense responses in plants under either biotic or abiotic stress, but also induces oxidative damage to plants^22^.

The variance in the effect of NO on root apoplastic Fe accumulation under different Fe supply conditions prompted us to investigate its underlying mechanisms. Several studies suggested that the level of polysaccharides in cell wall may be a factor affecting the amount of metal ion accumulation in root apoplast^23^,^24^. However, our results showed that GSNO treatment had little effect on the contents of pectin, HC1 and HC2 irrespective of Fe supply conditions (Fig. 5), indicating that both the increase of apoplastic Fe in Fe-deficient roots and the decrease of apoplastic Fe in Fe-sufficient roots should not be attributed to a change of polysaccharide level in the cell wall. Interestingly, here we found that Fe-deficient roots and Fe-sufficient roots had the same changes in Fe storage in different cell wall components upon GSNO treatment, i.e., the level of Fe in pectin fraction increased, while that in HC1 and HC2 fractions decreased (Fig. 4). Therefore, alteration of root-apoplastic Fe by NO may be due to its regulation in Fe storage in different cell wall components. However, a question was raised as to how the same regulation of Fe storage in different cell wall components by NO leads to contradictory alterations in apoplastic Fe accumulation in Fe-deficient roots and Fe-sufficient roots (Fig.2; Supplementary Fig. S3). This may be attributed to the following three reasons: (1) the concentrations of Fe-HC1 and Fe-HC2 in the cell wall extracted from Fe-sufficient roots were much higher than those in the cell wall extracted from Fe-deficient ones; (2) both Fe-HC1 and Fe-HC2 fractions in Fe-sufficient roots were greatly decreased by GSNO treatment, whereas those Fe fractions in Fe-deficient roots were decreased only slightly by the same treatment; and (3) upon GSNO treatment, the sum of decreases in Fe-HC1 and Fe-HC2 fractions was greater than the increase of pectin Fe in Fe-sufficient roots, whereas the opposite was true in Fe-deficient roots.

Since the NO-increased apoplastic Fe immobilization in roots of Fe-deficient plants was mainly due to the increase of Fe retention in pectin, the underlying mechanism of this process merits further exploration. Pectin is secreted in a highly methyl-esterified form from the symplast into the apoplast, where de-methylation takes place by pectin methylesterase (PME)^25^. De-methylation of pectin in the apoplast resulted in free carboxylic groups, which can, in theory, provide more binding sites in the apoplast^26^. Recently, in rice bean (*Vigna umbellata*) plants, Zhou *et al.*^27^ demonstrated that elevation of NO level increased Al retention in root cell wall by decreasing the degree of methyl-esterification (DM) of pectin, whereas Fe and Al have the same valence in the cell wall. Therefore, variation in the DM of pectin in cell wall could be another important factor determining the binding capacity of cell wall for metal ions^28^. Interestingly, we observed that GSNO application clearly decreased the DM of the pectin in root cell wall under both Fe-deficient and Fe-sufficient conditions (Fig. 6d; Supplementary Fig. S4). Comparing the Fe concentration in pectin fraction, an inverse correlation between the Fe concentration and degrees of methyl-esterification of the pectin can be drawn under both Fe supply conditions. Actually, for pectin a closely negative relationship between DM of pectin and the content of metal ions (such as Al and Zn) in cell wall has been previously reported^29^,^30^. Therefore, the higher retention of Fe in root apoplast under Fe deficient conditions seems to be associated with the lower degree of pectin methyl-esterification. In accordance with the changes of degree of methyl-esterification of the pectin, a significantly increased of PME activity treated with GSNO compared with control treatments were observed both under Fe-deficient and Fe-sufficient conditions (Fig. 7). Therefore, we conclude that NO increased the negative charged sites in cell wall by stimulating the PME activity, which in turn reduced the DM of the pectin in root cell wall.

Whether such alteration in cell wall could influence the interaction between cell wall and Fe, hence its reutilization? In this study, we found that the cell wall material from tomato roots treated with GSNO had a higher adsorption capacity, but a lower desorption capacity than the cell wall from the control regardless of Fe supply (Fig. 8). Similarly, cell wall from Fe deficiency also showed a higher adsorption capacity, but a lower desorption capacity than that of Fe sufficient treatment, further confirming the involvement of NO in this process (Fig. 8). These results indicated that increased binding capacity between Fe and cell wall triggered by NO might responsible for the higher accumulation of Fe in root apopalst.

In conclusion, although previous studies shown that NO was an important chemical signal in regulating Fe-deficiency-induced responses in plants, the present study provided evidence that elevation of NO may be an unfavorable factor for the improvement of plant Fe nutrition under Fe-limited growth conditions, and physiological mechanisms underlying this process may involve increased PME activity caused by the elevated NO, which may result in a lower degree of pectin methylation of root cell wall, and thus increased binding ability of cell wall with Fe under Fe-deficient conditions. Consequently, Fe retention in root apoplast of Fe-deficient plants is enhanced, preventing Fe translocation from roots to shoots.

## Methods

### Plant culture

The tomato (*Solanum lycopersicum* cv. Micro-Tom) seeds were germinated in 0.5 mM CaSO_4_ solution. Twenty days after sowing, seedlings of similar size were transferred to 1 L pots (four holes per seedling holder, and one seedling per hole) filled with aerated, full-strength complete nutrient solution. The nutrient solution had the following composition (in μM): NaH_2_PO_4_ 250, MgSO_4_ 500, KNO_3_ 1,000, Ca(NO_3_)_2_ 500, H_3_BO_3_ 10, MnSO_4_ 0.5, ZnSO_4_ 0.5, CuSO_4_ 0.1, (NH_4_)_6_Mo_7_O_24_ 0.1, Fe-EDTA 50. The solution pH was adjusted to 6.5 using 1 M NaOH. The nutrient solutions were renewed daily. All plants were grown in controlled-environment growth chambers at a humidity of 70%, with a daily cycle of a 28 °C, 14-h day and a 22 °C, 10-h night. The daytime light intensity was 180 μMol photons m^−2^ s^−1^. After 8 d of growth in the complete nutrient solution, one-half of the plants were transferred to an otherwise identical nutrient solution with 1 μM Fe-EDTA, and another one-half of the plants were continuously cultured in the 50 μM Fe-EDTA containing nutrient solution. For experiments with GSNO treatment, 100 μM GSNO was added to the +Fe (50 μM) or –Fe (1 μM) system. For experiments with cPTIO treatment, an equimolar level to that of GSNO was used in 50 mL vials.

### Chemicals

GSNO was synthesized as reported previously by Stamler and Loscalzo^31^.

DAF-FM DA was purchased from Beyotime Institute of Biotechnology (http://www. beyotime.com/), cPTIO from Sigma (http://www.sigmaaldrich.com/), Glutathione from Aladdin (http://www.aladdin-reagent.com/).

### *In situ* measurement of NO in root

Nitric oxide was imaged using diaminofluorescein-FM diacetate (DAF-FM DA). The DAF-FM DA has been successfully used to detect NO production in both plants and animals. Roots (5 mm from root tip) were loaded with 10 μM DAF-FM DA in 20 mM HEPES/NaOH buffer (pH 7.4) for 30 min, washed three times in fresh buffer, and observed under a Nikon Eclipse E600 epifluorescence microscope equipped with a Nikon B-2A filter block (450–490 nm excitation filter, 505 nm dichroic mirror, 520 nm barrier filter). A 100 W high-pressure mercury-vapour lamp was used as the light source (HB-10103AF-Hg, Nikon, Tokyo, Japan). Exposure settings were constantly maintained during the fluorescence microscopy. The signal intensities of green fluorescence in the images of the young roots were quantified according to the method of Guo and Crawford^32^ by measuring the average pixel intensity with a Photoshop software (Adobe Systems, San Jose, California, USA). Data is presented as the means of fluorescence intensity.

### Cell wall fraction preparation and polysaccharide content measurement

The entire root systems of Fe-sufficient or Fe-deficient plants were harvested and washed in 0.5 mM CaSO_4_ for 15 min. Cell walls were extracted according to Zhong and Läuchli^33^. Briefly, roots were ground with a mortar and pestle in liquid nitrogen and then homogenized with 75% ethanol for 20 min in an ice-cold water bath. The sample was then centrifuged at 8,000 rpm for 10 min and the supernatant was removed. The pellets were homogenized and washed with acetone, methanol: chloroform at a ratio of 1:1, and methanol, respectively, for 20 min each, with each supernatant removed after centrifugation between the washes. The remaining pellet, i.e., the cell wall material, was freeze dried overnight and stored in a refrigerator at 4 °C for further analysis.

The prepared crude cell wall was fractionated into three fractions: pectin, hemicellulose 1 (HC1), and hemicellulose 2 (HC2) according to Yang *et al.*^34^. The pectin fraction was extracted twice with 0.5% ammonium oxalate buffer containing 0.1% NaBH_4_ (pH 4.0) in a boiling water bath for 1 h and pooling the supernatants (pectin). Pellets were subsequently subjected to triple extractions with 4% KOH containing 0.1% NaBH_4_ for 24 h and pooling the supernatants (HC1). The resulting pellets further extracted with 24% KOH containing 0.1% NaBH_4_ for 24 h and pooling the supernatants (HC2), and the residue was considered the cellulose fraction.

The uronic acid content in each fraction was assayed according to Blumenkrantz and Asboe-Hansen^35^. Briefly, 5 mL 98% H_2_SO_4_ (containing 0.0125 M Na_2_B_4_O_7_·10H_2_O) was added to the 200 μL fraction extract in an ice water bath, followed by mixing and incubation at 100 °C for 5 min, and then cooling in the ice water bath. After cooling, 100 μL M-hydro-diphenyl (0.15%) was added to the solution and then the samples were incubated at room temperature for 20 min before absorbance at 520 nm was measured spectrophotometrically. Galacturonic acid was used as a calibration standard and the root total pectin, HC1 and HC2 concentrations were expressed as galacturonic acid equivalents.

### Fe content determination

To analyze the iron content, samples of leaves and roots were separately harvested from plants which were grown in the corresponding nutrient solutions for 7 d. After drying at 80 °C for 72 h, dry weights (DWs) were determined. The dried root or shoot samples were wet digested in concentrated HNO_3_ at 120 °C until there was no emission of brown NO gas, and then were further digested in HNO_3_/HClO_4_ at 180 °C until the solution became transparent. Digestates were diluted with ultrapure water and the concentration of Fe in the digestates was measured by inductively coupled plasma-mass spectrometry (ICP-MS) (Agilent 7500a, Agilent, Santa Clara, California, USA).

The total Fe concentration in cell wall was measured according to Lei *et al.*^36^. Briefly, the dry cell wall was suspended in 5 mL 2 M HCl at room temperature for 48 h with occasional shaking. After centrifugation, the supernatant was collected for Fe content analysis using ICP-MS. The pectin, HC1 and HC2 fractions were prepared and their Fe contents were measured by ICP-MS as well.

### Analysis of apoplastic Fe

The apoplastic Fe content was analyzed according to the method of Bienfait *et al.*^7^. Briefly, roots were transferred to a beaker containing 150 mL of aerated 0.5 mM CaSO_4_ for 15 min. Then the roots were placed in a test tube containing 150 mL of 1.5 mM 2.2-bipyridyl. Oxygen was displaced from the solution by bubbling with N_2_ (5 min) before and after addition of 12.5 mM Na_2_S_2_O_4_. Finally, an aliquot of the solution containing the Fe(II)-bipyridyl complex was measured spectrophotometrically at 520 nm.

### Xylem sap sampling

Tomato xylem sap was sampled using the de-topping technique according to López-Millán *et al.*^37^. Plant shoots were cut just below the first true leaf using a razor blade, and xylem sap was left to exude. The sap of the first 5 min was discarded to avoid contamination. The surface of the opening was washed with distilled water and blotted dry, and the sap was then collected for 40 min using a micro-pipette and maintained in Eppendorf tubes kept on ice. Xylem sap was pooled from four plants for one biological replicate. The total volume was determined and the samples were stored at −80 °C until further analysis. Before analysis, samples were thawed and diluted with dilute nitric acid, and the Fe concentration was determined by inductively coupled plasma-mass spectrometry.

### Analysis of Fe adsorption and desorption kinetics

The Fe adsorption and desorption kinetics were analyzed as described earlier in Jin *et al.*^5^ and Zheng *et al.*^38^. Briefly, a total of 10 mg cell wall powder was weighed into a 2 mL column fitted with a filter at the bottom. The adsorption solution consisted of full-strength complete nutrient solution and the desorption solution consisted of Fe-omitting full-strength nutrient solution. The solution was sipped by a peristaltic pump at a speed of 4 mL·20 min^−1^ passing through the column and collected at 20 min intervals into tubes on a fraction collector. The Fe in the adsorbed solutions was measured with 2,2-bipyridyl according to the method of Bienfait *et al.*^7^. The unbound Fe was washed with 0.5 mM CaCl_2_ at pH 6.5 at a speed of 10 mL·min^−1^ for 1 h. Then the bound Fe was desorbed by Fe-free full-strength nutrient solution at 2 mL·20 min^−1^, and the fraction was collected until the Fe concentration in the desorption solution was below the detection limit.

### Pectin methylesterase activity assay

Pectin methylesterase (PME) in root was extracted using a high-salt buffer as described in Ren and Kermode^39^. Roots of tomato were placed in the PME extraction buffer (0.1 M citrate acid, 0.2 M Na_2_HPO_4_, and 1 M NaCl, pH 5.0), and were fully ground at 4 °C. The homogenized slurry was transferred to an Eppendorf tube, and then was incubated on ice for 1 h, during which it was vibrated three times at 20 min intervals, and centrifuged for 10 min at 15,000 × g at 4 °C. The supernatant was collected for further analysis. The PME activity was examined according to the method described by Richard *et al.*^40^. The extract was added to the PME activity assay buffer (0.5% citrus pectin, 0.2 M NaCl, and 0.015% methyl red, pH 6.8) and the mixture was incubated for 1.5 h at 37 °C. Pectin de-esterification decreases the pH, thus changing the color from yellow to red. The color change was recorded at 525 nm with a spectrophotometer. A calibration curve was obtained by adding 10-300 μL 0.01 M HCl to the 4 mL PME activity assay buffer and the respective optical density (OD) values were measured at 525 nm.

### Determination of the degree of methyl-esterification (DM) of pectin

The DM of pectin was spectrophotometricly determined as described in Klavons and Bennett^41^ Pectin methyl ester was hydrolyzed as follows: 10 mL of 1 M KOH was added to aliquots of the pectin fraction to give 15 mL of pectin solutions. The solutions were incubated at room temperature for 40 min. The pectin hydrolysates were neutralized with dilute phosphoric acid to pH 7.5 and adjusted to 20 mL with ultrapure water. 1 mL hydrolyzed pectin samples were mixed with 1 mL alcohol oxidase (1 UN·mL^−1^) and incubated at 25 °C for 15 min. Then, 2 mL of fluoral-P (0.02 M 2,4-pentanedione in 2.0 M ammonium acetate and 0.05 M acetic acid) was added and vortex mixed. The mixtures were incubated at 60 °C for 15 min then cooled to room temperature and analyzed for methanol. The absorbance was measured at 412 nm against a blank. Methanol was used for the calibration curve. The pectin hydrolysates were analyzed for uronic acid as described previously.

Alternatively, the DM of pectin also measured by Fourier transform infrared (FTIR) spectroscopy as Manrique and Lajolo^42^. About 1 g (DW) of cell wall material was extracted three times with 0.5% ammonium oxalate buffer containing 0.1% NaBH_4_ in a boiling water bath for 1.5 h, the supernatants were collected and combined then dialysis against distilled water, the extract were freeze-dried, then desiccated in an oven for 1 week at 30 °C and transferred to vacuum jar containing silica gel before analysis. Dry samples were analyzed with FTIR spectroscopy (Nicolet 6700, Thermo Fisher Scientific, Waltham, Massachusetts, USA). The spectra were recorded at the absorbance mode from 4000 to 400 cm^−1^ at a resolution of 4 cm^−1^ with 128 co-added scans. The DM was calculated using the absorbance intensities at 1630 and 1740 cm^−1^ assigned to the carbonyl groups (COO^-^) of GalA and its methylester, respectively, with the equation described by Manrique and Lajolo^42^. DM = A_1740_/(A_1740_+A_1630_) × 100%. Commercial pectins with different determined DM (29.97%, 60.92% and 89.78%) were used as standards purchased from Sigma.

### Statistical analysis

The data were subjected to analysis of variance (ANOVA), and the least significant difference (LSD) test was employed to determine differences among the treatments at *P* = 0.05 levels.

## Additional Information

**How to cite this article**: Ye, Y. Q. *et al.* Elevation of NO production increases Fe immobilization in the Fe-deficiency roots apoplast by decreasing pectin methylation of cell wall. *Sci. Rep.* **5**, 10746; doi: 10.1038/srep10746 (2015).

## Supplementary Material

Supplementary Information
