# Supplementary material for: Elevation of NO production increases Fe immobilization in the Fe-deficiency roots apoplast by decreasing pectin methylation of cell wall
Source: Sci Rep. 2015 Jun 15;5:10746. doi: 10.1038/srep10746 (PMC4466582; doi:10.1038/srep10746)

Supplementary Information

Title:

Elevation of NO production increases Fe immobilization in the Fe-deficiency roots apoplast by decreasing pectin methylation of cell wall

Authors:

Yi Quan Ye[1a], Chong Wei Jin[1,2a], Shi Kai Fan[1], Qian Qian Mao[1], Cheng Liang Sun[1], Yan Yu[1], Xian Yong Lin[1,2*]

[1]MOE Key Laboratory of Environment Remediation and Ecological Health, College of Natural Resource & Environmental Sciences, Zhejiang University, Hangzhou 310058, China;

[2] Key Laboratory of Subtropical Soil Science and Plant Nutrition of Zhejiang Province, College of Environmental and Resource Sciences, Zhejiang University, Hangzhou 310058, PR China.

[a] these authors contributed equally to this work and paper.

* To whom correspondence should be addressed. E-mail address: xylin@zju.edu.cn

Figure legends

Fig. S1 Apoplast iron of Arabidopsis thaliana wild-type (wt) and *gsnor1-3* mutant plants under iron deficiency. 30 d old seedlings were exposed to Fe-deficient (1 μM Fe) solution for 7 days and then root apoplast iron was analyzed. Error bars represent ±SD (n=5). Bars with different letters are significantly different at $P < 0.05$.

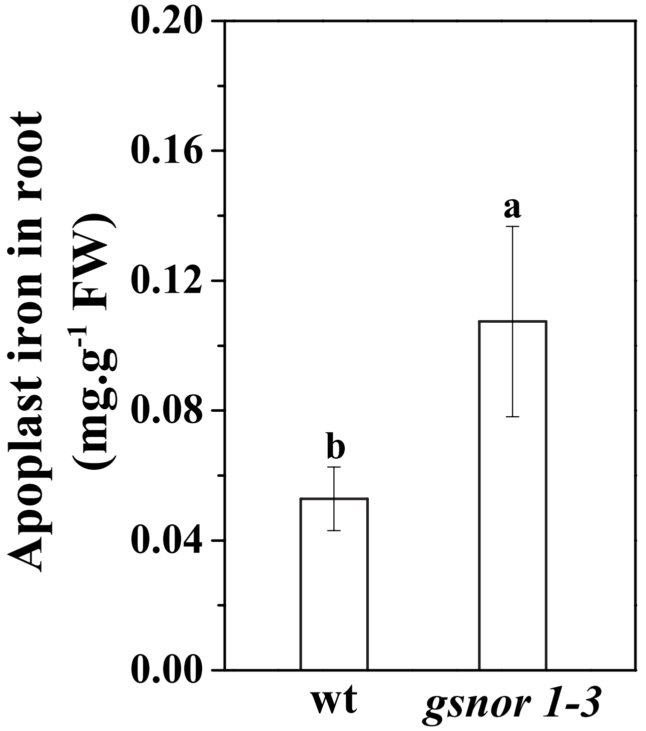

Fig. S2 NO production in root of tomato plants treatment with NO donor or scavenger under Fe sufficient conditions. Photographs of NO production shown as green fluorescence in representative roots (a) (bar=1 mm). NO production expressed as relative fluorescence (b). The seedlings were grown under Fe sufficient (50 μM Fe) conditions treated with or without 100 μM GSNO or 100 μM cPTIO for 7 days. The roots of plants after 7 days of treatment were harvested for NO analysis. Data are means ±SD (n=15). Bars with different letters are significantly different at $P < 0.05$

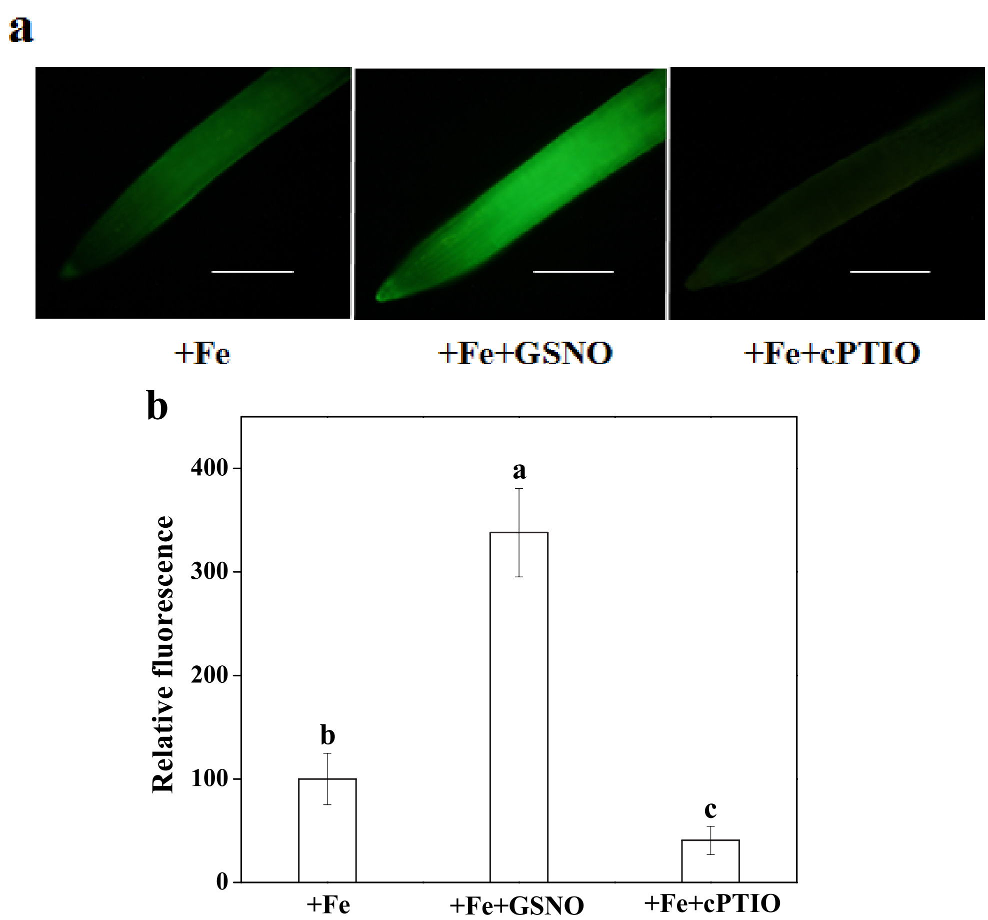

Fig. S3 Effect of NO on Fe concentrations in root apoplast of tomato plant under Fe sufficient conditions (50 μM Fe). The seedlings were grown under Fe sufficient (50 μM Fe) conditions treated either with 100 μM GSNO or 100 μM GSNO plus 50 μM cPTIO for 7 days and Fe concentrations in root apoplast were analyzed. Error bars represent ±SD (n=4). Bars with different letters are significantly different at $P < 0.05$

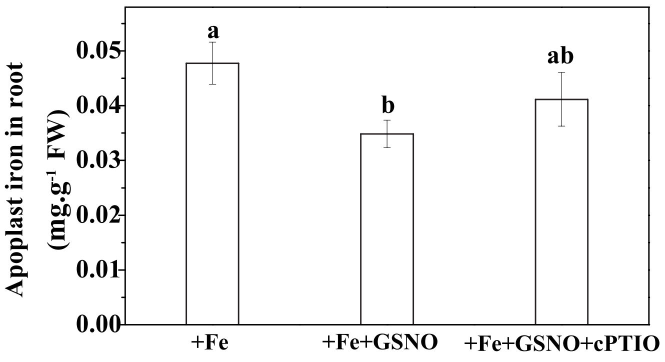

Fig. S4 Effect of NO on the degree of methyl-esterification (DM) of the pectin. The seedlings were treated with or without 100 μM GSNO for 7 days under Fe deficient (1 μM Fe) or Fe sufficient (50 μM Fe) and then the degree of pectin methylation was analyzed. Error bars represent ± SD (n=5). Bars with different letters are significantly different at $P < 0.05$.

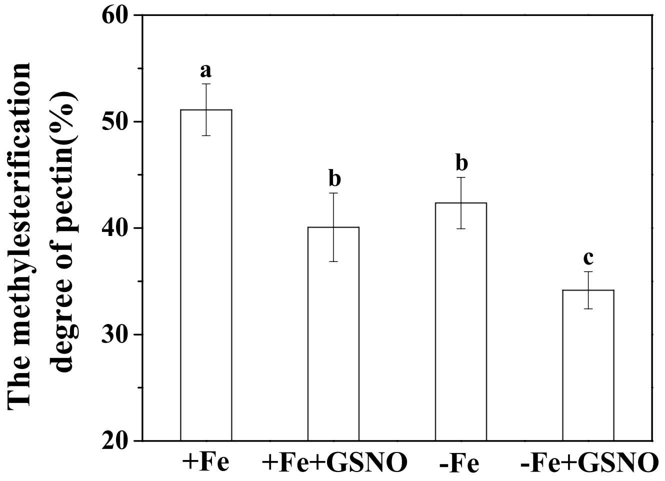

Supplement: Supplementary Information [file srep10746-s1.pdf]
